# Onco@home: comparing the costs and reimbursement of cancer treatment at home with the standard of care

**DOI:** 10.1186/s13690-024-01317-1

**Published:** 2024-06-24

**Authors:** Sarah Misplon, Wim Marneffe, Jana Missiaen, Dries Myny, Inge Decock, Steve Lervant, Koen Vaneygen

**Affiliations:** 1 Faculty of Business Economics, Hasselt University, Hasselt, Belgium; 2 AZ Groeninge, Kortrijk, Belgium; 3 OLV Lourdes, Waregem, Belgium; 4 Sint-Jozefskliniek, Izegem, Belgium; 5 Department of Public Health and Primary Care, Ghent University, Ghent, Belgium

**Keywords:** Cost analysis, Micro-costing, Health insurance, Home Hospitalization

## Abstract

**Background:**

Oncological home hospitalization (HH) was implemented in a Belgian context to evaluate the feasibility of oncological HH. In a first HH model (HH1), implemented by three Belgian hospitals, two home nursing organizations and a grouping of independent nurses, the blood draw and monitoring prior to intravenous therapy was performed by a trained home nurse at the patient’s home the day before the visit to the day hospital. In a second HH model (HH2), implemented in one hospital, the administration of two subcutaneous treatments (Azacitidine and Bortezomib) for myelodysplastic syndrome and multiple myeloma were provided at home instead of in the hospital. A previous study on this pilot showed that oncological HH is feasible and safe and improves the Quality of Life. The aim of this study is to investigate the cost and reimbursement of cancer treatment in these two HH models compared to the Standard of Care (SOC).

**Methods:**

A bottom-up micro-costing study was conducted to compare the costs and revenues for the providers (hospitals and home care organizations) of the SOC and the HH models.

**Results:**

Costs associated to HH were higher than the SOC in the hospital. Comparing revenues with costs, the research revealed that the reimbursement from the National Health Insurance of HH for oncological patients is insufficient. In HH1, costs were higher than in the SOC (+ €50.4). There was a reduction in costs in the hospital by moving the blood draw to the home setting (-€23.9), but the costs in home care were higher (+ €74.3). The extra revenues in home care (+ €33.6) were insufficient to cover the costs. The cost difference between the SOC and HH2 (+ €9.5 for Azacetidine) was smaller than in HH1. But, there was almost no funding for subcutaneous administration in home care. If the product is administered in a day hospital, the hospital receives a revenue of €124 per administration, while in home care the funding is €5 per visit.

**Conclusion:**

Costs of HH are higher and the reimbursement from Belgian NHI is insufficient to organize HH. As a result, HH for oncology patient is still limited in Belgium.

**Supplementary Information:**

The online version contains supplementary material available at 10.1186/s13690-024-01317-1.

**Table:** 

Textbox 1. Contributions to the literature	
• This study is part of a larger research project to evaluate the feasibility of oncological HH in a Belgian context. Therefore, two HH models were implemented during a pilot period (2016–2022).	
• Existing research has indicated a scarcity of information regarding the financial implications of HH models [3, 5].	
• The costs are calculated using a micro-costing approach, a method of cost estimation that relies on comprehensive data regarding resource consumption and unit costs.	

## Background

Cancer care is evolving rapidly, with advances in treatment and increasing healthcare costs [13]. In the European Union, cancer care is responsible for 6.2% of all healthcare expenditure (Hofmarcher, 2019). Over the last two decades, health spending on cancer care has increased more rapidly than cancer incidence. [9].

Home hospitalization (HH) is a possible approach to offer high-quality, patient-centered care and create value for patients. Alves et al. [1] defined HH as “a service that provides active treatment by healthcare professionals in the patient’s home for a condition that otherwise would require acute hospital inpatient care, and always for a limited time period.”

This study is part of a larger research project to evaluate the feasibility of oncological HH in a Belgian context. The goal of this research project is to learn from the practical implementation of HH, to elaborate a roadmap for implementation, and to advise the government on the legal, financial, and other barriers and opportunities. Therefore, two HH models were implemented during a pilot period (2016–2022) with the support of a social profit organization. In a first HH model (HH1), implemented by three hospitals, two home nursing organizations and a grouping of independent nurses, the blood draw and monitoring prior to intravenous therapy was performed by a trained home nurse at the patient’s home the day before the visit to the day hospital. In a second HH model (HH2), implemented by one hospital, the administration of two subcutaneous treatments (Azacitidine and Bortezomib) for myelodysplastic syndrome and multiple myeloma was provided at home instead of in the hospital.

In reviewing the literature, as part of this research project, Cool et al. [5] found that a large majority of HH patients are satisfied with HH (12/13 studies) and prefers home treatment (7/8 studies). The review also revealed that HH might be considered as safe and has no significant effect on the reported Quality of Life (7/8 studies).

Subsequently, a Randomized-Controlled Equivalence Trial with a total of 148 participants (n = 74 in each group) was conducted, confirming the viability and safety of the implementation of oncological HH while having no discernable effect on patient-reported Quality of Life. HH1 led to a significant reduction of waiting time before therapy administration at the day care unit by 45% per visit (2 h 36 min ± 1 h 4 min vs. 4 h ± 1 h 4 min; P < 0.001). In total, 88% of the intervention group reported high levels of satisfaction with HH practices, while 77% reported a positive impact on their Quality of Life. Ultimately, 60% of participants in both groups opted for HH as the preferred intervention over standard of care. [3, 4]

However, little information is available on the costs of HH. The systematic review of Cool et al. [5] revealed that only five studies compared the costs for oncological HH to the costs in the hospital. These studies considered different cost perspectives, including the national health insurance (NHI), the provider, and society, making cross-study comparisons challenging. In examining provider costs, King et al. [10] demonstrated that providing chemotherapy at home is more expensive than in-hospital care, primarily due to increased nursing time in HH. Similarly, in a more recent study, Franken et al. [6] found higher healthcare costs associated with home-based administration of subcutaneous trastuzumab, also because of increased nursing time (110 min for HH versus 38 min in a hospital setting). Rischin and Matthews [14] also reported increased costs associated with HH treatments. On the other hand, Lüthi et al. [11] concluded that home care results in a 53% cost benefit compared to hospital treatment. None of the cited studies compared the production costs for the provider with the reimbursement from national health insurance (NHI) while reimbursement is a key success factor in the uptake of HH [7].

The researchers [3, 5] concluded that further research on the financial impact of HH models is needed. Therefore, the first objective of this study is to investigate the costs of the implemented HH models compared to the SOC. The second objective is to investigate whether the current reimbursement from the NHI of HH for oncological patients is sufficient to cover the costs for the providers (hospitals and home care organizations).

## Methods

### Scope

In this study, cost and revenues of oncology care were calculated from a providers’ perspective for the standard ambulatory hospital care and for two HH models. The study focused on the hospital and home nursing costs. Doctors’ activities were excluded from the study as medical doctors are independent and are reimbursed separately via a distinctive funding model. Additionally, pharmaceutical expenditures were excluded from the study as they vary widely based on the therapy utilized and are reimbursed according to a separate system. Finally, patient and family costs were ignored due to the provider-perspective used.

### Patients

Patients were eligible for home hospitalization in this study if they were 18 years or older, possessed a good performance status (Eastern Cooperative Oncology Group ≤ 2), resided within a 30-min drive from the hospital, and had a diagnosis of either a solid tumor or hematologic malignancy necessitating the initiation or continuation of active treatment, whether curative, palliative (i.e., noncurative treatments), or supportive (e.g., blood transfusions), at the oncology day care unit (DCU). Exclusion criteria encompassed patients with problematic venous access, known issues with therapy administration, simultaneous radiotherapy treatment < 12 weeks of planned therapy, language barriers, or communication difficulties.

### Design

To calculate the costs, a bottom-up micro-costing study was conducted for the providers (hospitals and home care organizations). Bottom-up micro-costing is described by Tan et al. [17] as the gold standard methodology for the costing of hospital services. Costs were calculated as described in the Belgian manual for cost-based pricing of hospital interventions, elaborated by the Belgian Health Care Knowledge Centre (Swartenbroeckx et al. [15, 16]). The average cost per care pathway was calculated for (1) staff, (2) materials, (3) traveling, and (4) other costs (cleaning, heating, linen, catering, administration, general depreciation costs, etc.).

Revenues were gathered from the Belgian National Institute for Health and Disability Insurance (NIHDI). The Belgian hospital financing system for oncology day care patients consists of different elements (Van de Sande et al. [18], Van de Voorde et al. [19]): (1) A lump sum for nursing activities for preparation of patients, interventions as well as for after-care costs, costs of bedding and laundry, cleaning, heating; (2) A fee for service system for doctors’ procedures (consultations, lab tests, radiology); (3) The reimbursement of pharmaceuticals, including chemotherapy. For the revenues in the hospital, only the lump sum for nursing activities was taken in scope of this study, as doctors’ costs and pharmaceuticals were also excluded from the cost calculation. The lump sum specific for the admission of chemotherapy varies according to the admission of one or multiple products per visit. In home care, home nurses work in a fee for service system. Those fees were collected for the home hospitalization activities for HH1 and HH2.

## Study intervention

**Fig. 1:**
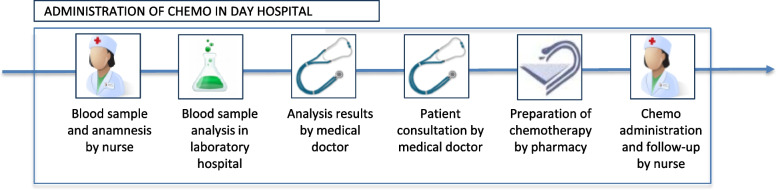
Standard of care

The standard ambulatory hospital care process (SOC) entails the patient's arrival at the day hospital, where all necessary medical procedures are conducted. These include sample collection for blood analysis and anamnesis by an oncology nurse, blood analysis, data interpretation by the physician, consultation with the physician, preparation and administration of chemotherapy and follow-up, illustrated in Fig. 1.

**Fig. 2:**
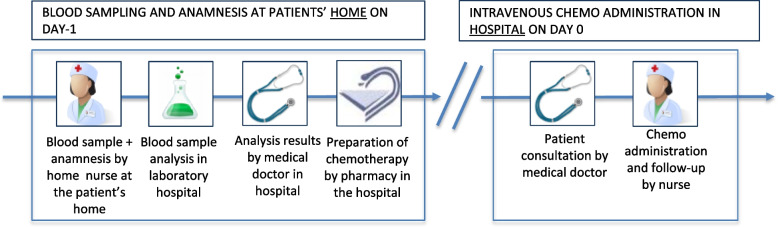
Home hospitalization model 1: Performing preparation in the home environment prior to intravenous therapy at the day hospital the next day

In a first HH model (HH1), blood draw and anamnesis before intravenous therapy was performed by a trained home nurse at the patient’s home the day before the hospital visit. By conducting these assessments one day prior to therapy administration (i.e., on day -1), oncologists were able to prescribe therapy and the pharmacy department could prepare treatments in advance of patient arrival at the hospital, which reduces the waiting time before the administration of chemotherapy [3], see Fig. 2. The prerequisite is that the treatment can be prepared in advance, which is the case for more than 95% of al treatments.

**Fig. 3:**
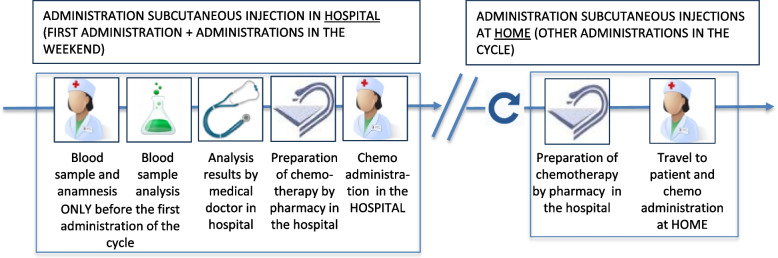
Home hospitalization model 2: Subcutaneous administration of chemotherapy in the home setting

In a second HH model (HH2) (see Fig. 3), the administration of two subcutaneous treatments (Azacitidine and Bortezomib) for myelodysplastic syndrome and multiple myeloma are provided partly at home instead of in the hospital. In this study, the first administration per cycle and the administrations in the weekend are performed in the hospital. The other administrations are executed at the patient’s home, after performing a telephone symptom burden survey. Those two treatments were selected because of the frequent visits to the hospital per treatment and the burden this brings for patients. Because of the financial barriers (explained further), only one hospital decided to participate in this HH2. In this hospital a hospital nurse administers the treatment at home.

## Care pathways included in the calculation

**Fig. 4:**
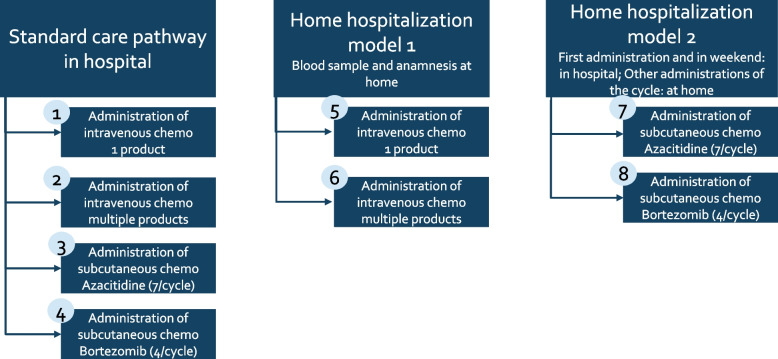
Overview of the types of patients identified for the cost calculation

For the cost and revenue calculation, a further subdivision in eight different types of care pathways was made, see Fig. 4.

In the SOC, a distinction was made between two types for the administration of intravenous chemo and two types for the administration of subcutaneous chemo. For intravenous chemo, the administration of one or multiple chemo products per day has an impact on the administration time. Also, the reimbursement for the administration of one product is lower than for the administration of multiple products.. In the cost calculation, the average costs and revenues of one visit to the day care center was calculated.

For subcutaneous treatment, a distinction was made between the subcutaneous administration of Azacitidine, with seven administrations per cycle, and the subcutaneous administration of Bortezomib, with four administrations per cycle. (See Fig. 4: Care Pathways 3 and 4). In the cost calculation, for Care Pathway 3 the average cost of one visit, 7 visits per cycle, one blood test per cycle was calculated. In Care Pathway 4 the average cost of one visit, four visits per cycle, one blood test per cycle was included in the cost calculation.

In HH1, a division in two groups is made: the administration of one product and the administration of multiple products, as in the SOC. In the cost calculation, the average costs and revenues per visit for each care pathway were calculated. Therefore, the cost and revenues of the blood analysis at home performed by a home care organization and the administration of chemo in the hospital were included.

In HH2, as in the SOC group, there was also a distinction made between the administration of Azacitidine (Care Pathway 7) and Bortezomib (Care Pathway 8). In Care Pathway 7 the average of one visit was calculated, based on 7 visits per cycle: three in the hospital and four at home performed by hospital oncology nurse at home and one blood test per cycle. In Care Pathway 8 the average of one visit was calculated, based on four visits per cycle: one in the hospital and three at home performed by a hospital oncology nurse at home and one blood test per cycle.

### Data collection

Cost and revenue data for the year 2019 were gathered, as 2020 and 2021 were impacted by COVID in terms of costs. Data were collected in three hospitals for the SOC, in three hospitals and in two home care organizations for HH1 and in one hospital for HH2.

### Costs

To calculate the staff costs, the average time and the cost per minute of each activity were calculated. Therefore, activities of the different care pathways were mapped in the three hospitals and two home care organizations. Subsequently, time registrations per activity were performed during the period between October 2020 and April 2021. In this period, there was a normal activity for oncological patients. Even though it was still during the COVID-pandemic, chemotherapy was delivered to patients in the hospital as before COVID.

To calculate the time per activity in the day care units of the three hospitals, nurses were followed by a researcher during 12 days, 4 in each hospital, and the time of every activity and patient was registered. To simplify the data collection process, an excel macro was built with buttons to indicate at the start of each activity, after which the start time was automatically recorded (see supplementary information – figure A). To take all time into account, also the time not related to a patient of the followed nurses was registered (e.g. walking, administration, and logistical tasks).

In two hospitals, because of the architecture and scale of the day care unit, it was possible for the researcher to register the time of all nurses active in the unit between 7 AM and 6 PM. In one larger hospital, only one nurse per day was followed during a full shift. In that hospital, 3 early shifts (from 7 AM to 4 PM) and one late shift (from 9 AM to 6 PM) were followed. For each activity directly related to a patient, the patient and room number was registered. After every registration day, all followed patients were allocated to the different care pathways by the researcher together with an oncology nurse of the department. Not all patients could be allocated to one of the defined care pathways, as the day care unit also performs other treatments. In total, time registrations of 46 patient visits were included.

In HH1, the home care nurses and administrative staff of two home care organizations self-registered the time of every direct and indirect activity during 2 weeks between September and December 2020. In total, time registrations of 99 patients were collected. In the supplementary information the registration form with the list of activities is included.

HH2 was only implemented in one hospital on a limited number of patients. The visits to the patients were performed by an oncology nurse of the oncology day care center. In HH2 the oncology nurse performed time registrations of all direct and indirect time during 2 weeks. In total, time registrations of 15 HH2 patients were gathered during the period September–October 2020. In the supplementary information (table A) the registration form with the list of activities is included.

The time not related to a patient of the followed nurses was added to the direct care time as a percentage of the direct care time per visit.

Subsequently, a cost-per-minute-per-profile was calculated. To do this, wage costs from the accountancy department of the three hospitals and two home care organizations were collected. To calculate the cost per minute, 1,605.2 h/year of productive time was used, as advised by the Belgian Federal Health Care Knowledge Center in their manual for cost-based pricing of hospital interventions (Swartenbroekx et al. [15, 16]).

For the material costs, the oncology nurses in two hospitals registered all materials necessary in the SOC, HH1, and HH2 and collected the costs of each material from their accounting departments.

To calculate the car costs, the nurses registered the distance to each patient during the time registration of HH1 and HH2. Based on this information, an average distance per patient was calculated. Per km an average cost of €0.35 was used, based on the kilometer allowance defined by the government (Federale Overheidsdienst Beleid en Ondersteuning, 2020). The average distance in HH1 was 9.46 km (± 3.9 km SD) and in HH2 11.33 km (± 6.4 km SD).

For the administrative, logistic and coordinating staff in the day hospital, the total cost per profile per hospital was requested. To calculate the average cost per patient and per hospital, the cost per year was divided by the total number of day hospital patients.

For general overhead costs in the hospital, a mark-up percentage of 56.6% on direct costs was used for maintenance, heating, linen, catering, administration, general depreciation costs, etc., as calculated in the Belgian manual for cost-based pricing of hospital interventions, based on accounting information of all Belgian hospitals (Swartenbroekx et al. [15, 16]). The accounting information of 2019 of the three involved hospitals in the study learns that the average overhead cost for the oncological day care hospital is in line with this percentage.

In the included home care organizations, the average overhead percentage for the costs of administration, buildings, management, was 13.2% mark-up on staff costs, based on information received by the accounting departments of the included home care organizations.

### Revenues

The average revenue per care pathway was calculated based on invoices and pricing information from NHI. Patient invoices of the year 2019 were collected in the hospitals (n = 4,669). In home care organizations, only a few activities (nomenclature numbers) can be charged to the NHI. These charges and their implementation rules were gathered during interviews with the nursing coordinators of the two home nursing organizations.

### Input parameters

All input parameters (unit cost per minute per profile, average time per activity and travel time in minutes per visit, material costs, travel costs, costs for coordination, logistics and administration and revenues) are included in the supplementary information.

### Assumption

In the cost calculation it was assumed that, in the short term, there would be no reduction of the overhead costs in the hospital per patient. The number of beds, m^2^, staff, and so on will not change because of this project.

## Results

The calculations of costs, revenues and financial results per care pathway are performed per type of care pathway (see Fig. 4). Based on the input parameters an average cost per visit per type of care pathway was calculated for the SOC, HH1 and HH2.

Table 1 gives the costs and revenues in the SOC.

**Table 1:** Calculation of costs and revenues per visit – SOC

Care pathway	Standard of care - Administration in hospital			
	Calculation base = 1 visit to the day care center			
	Average cost per visit			
Type	Intravenous treatment, 1 product	Intravenous treatment, multiple products	Subcutaneous treatment Azacitidine	Subcutaneous treatment Bortezomib
Number	1	2	3	4
Costs	€170.85	€220.87	€121.18	€122.65
Cost of care time nurses (see supplementary information – table E)	€65.37	€92.17	€42.12	€42.12
Cost of coordination, logistics and administration (see supplementary information – table F)	€33.60	€33.60	€33.60	€33.60
Material cost (see supplementary information – table I)	€10.13	€15.27	€1.66	€2.60
Hospital overhead costs (+ 56.6% on the standard care pathway)	€61.75	€79.83	€43.80	€44.33
Revenues	€124.10	€166.11	€124.10	€124.10
RESULT (Revenues – Costs)	-€46.75	-€54.76	€2.92	€1.45

Based on the performed calculations, we found that the SOC for intraveneous products is loss-making (-€46.75 for the administration of one product and -€54.76 for the administration of multiple products). For subcutaneous treatment, the result is slightly profitable (+ €2.92 for Azacitidine and + €1.45 for Bortezomib). The reason is that the revenues per visit are the same: a fixed fee of €124.10 for the administration of intravenous chemo 1 product or subcutaneous treatment and €166.11 for the administration of multiple products, while the administration of intravenous chemo is more time-intensive than the administration of subcutaneous treatment.

Table 2 gives the average costs and revenues per visit for HH1. In the cost calculation it was assumed that, in the short term, there would be no reduction of the overhead costs in the hospital per patient. The number of beds, m^2^, staff, and so on will not change because of this project. Therefore, cost of coordination, logistics, administration and overall overhead costs in the hospital were assumed to remain the same in HH1 and HH2.

**Table 2:** Calculation of costs and revenues per visit, HH1

Type	**Average cost per visit**	
	**Intravenous treatment, 1 product**	**Intravenous treatment, multiple products**
Number	**5**	**6**
Cost hospital + home care	€221.2	€271.30
Day care oncology unit	€147.00	€197.02
Cost of care time nurses (see supplementary information – table G)	€50.28	€77.08
Cost of coordination, logistics and administration (see supplementary information – table F)	€33.60	€33.60
Material cost (see supplementary information – table I)	€1.37	€6.51
Overhead costs (+ 56.6% on the standard care pathway)	€61.75	€79.83
Home care	€74.26	€74.26
Total staff costs (see supplementary information – table G)	€54.54	€54.54
Car costs (see supplementary information – table J)	€3.31	€3.31
Material cost (see supplementary information – table I)	€9.21	€9.21
Overhead costs (+ 13.2% on staff costs in HH1)	€7.20	€7.20
Revenues	€157.68	€199.69
Hospital	€124.10	€166.11
Home care	€33.58	€33.58
RESULT	- €63.58	- €71.59
Hospital	- €22.90	-€30.91
Home care	-€40.68	-€40.68

In Table 2, the costs and revenues of HH1 in the day care oncology unit and home care were calculated. The blood draw and symptom control is performed in home care. As a result there is a reduction of the care time and the cost of the nurses in the hospital. The cost of home care is calculated as €74.26. In home care, home nurses receive a fee of €33.58, which is not sufficient to cover the full cost of the transport, the care time, administration of the home care organization. Also, in the hospital, HH1 is still loss-making. As a result, both the admission of intravenous treatment of one (-€63.58) and of multiple products (-€71.59) is loss-making in total.

Table 3 gives the average costs and revenues per visit for HH2. In this calculation, the average of 1 visit is calculated. In home care, the fee is only €5.30 per visit, while this is €124.10 in hospital (See: Supplementary information – Table K: Revenues per visit).

**Table 3:** Calculation of average costs and revenues per visit, HH2

Type	**Average cost per visit**	
	**Subcutaneous treatment at home—Azacitidine** **3 visits in hospital, 4 at home**	**Subcutaneous treatment at home—Bortezomib** **1 visit in hospital, 3 at home**
Number	**7**	**8**
Cost hospital + home care	€130.68	€135.12
Day care oncology unit	€97.11	€91.06
Cost of care time nurses (see supplementary information – table G)	€18.05	€10.53
Cost of coordination, logistics and administration (see supplementary information – table F)	€33.60	€33.60
Material cost (see supplementary information – table I)	€1.66	€2.60
Overhead costs (+ 56.6% on the standard care pathway)	€43.80	€44.33
Administration at home by hospital oncology nurse	€33.57	€44.06
Total staff costs (see supplementary information – table G)	31.68	€41.58
Car costs (see supplementary information – table J)	€1.89	€2.48
Revenues	€56.22	€34.68
Hospital: (revenues of SOC * # visits in hospital) / total # visits per cycle	€53.19	€31.03
Home care: (revenues per visit * # visits in hospital) / total # visits per cycle	€3.03	€3.65
RESULT	- €74.46	- €100.44
Hospital	- €43.92	-€60.03
Home care	-€30.54	-€40.41

For Azacitidine, there are 7 visits per cycle: three in the hospital and four at home performed by a hospital oncology nurse at home and one blood test per cycle during the first visit to the hospital. The average result per admission is -€74.46. For Bortezomib, there were four visits per cycle: one in the hospital and three at home performed by a hospital oncology nurse at home, one blood test per cycle. The average result per admission is -€100.44.

As illustrated in Table 4 costs are overall higher in HH1 than in the SOC (+ €50.42). There is a reduction in costs in the hospital by moving the blood draw to the home setting (-€23.84), but the costs in home care are higher (+ €74.26). We also see that the revenues in home care are insufficient to cover the costs. In HH1, the revenues in home care are €33.58 per visit and in the hospital the revenues remain the same. As a result, the loss is €16.84 euro higher in HH1 than in the SOC.

**Table 4:** Comparison of HH1 and HH2 to the SOC

	**Difference HH 1 – SOC**		**Difference HH 2 – SOC**	
	**Intravenous treatment** **1 product**	**Intravenous treatment multiple products**	**Subcutaneous treatment** **Azacitidine**	**Subcutaneous treatment** **Bortezomib**
**Cost hospital + home care**	+ €50.42	+ €50.42	+ €9.50	+ €12.47
**Day care oncology unit**	-€23.84	-€23.84	-€24.07	-€31.59
**Total staff cost**	-€15.08	-€15.08	-€24.07	-€31.59
**Material cost**	-€8.76	-€8.76	+ €0.00	+ €0.00
**Overhead costs (+ 56.6% on of the standard care pathway (1))**	+ €0.00	+ €0.00	+ €0.00	+ €0.00
**Home care**	+ €74.26	+ €74.26	+ €33.57	+ €44.06
**Staff costs**	+ €54.54	+ €54.54	+ €29.2	€38.3
**Transport costs**	+ €3.31	+ €3.31	+ €1.9	€2.5
**Material costs**	+ €9.21	+ €9.21	+ €0.0	€0.0
**Overhead costs (+ 13.2% on staff costs)**	+ €7.20	+ €7.20	+ €0.0	€0.0
**Revenues, excluding lab tests and MD oncologist**	+ €33.58	+ €33.58	-€67.88	-€89.42
**Hospital**	+ €0.00	+ €0.00	-€70.91	-€93.07
**Home care**	+ €33.58	+ €33.58	+ €3.03	€3.65
**RESULT (Revenues – Costs), excluding lab tests and MD oncologist**	- €16.84	- €16.84	- €77.38	- €101.89
**Hospital**	+ €23.84	+ €23.84	- €46.84	- €61.48
**Home care**	-€40.68	-€40.68	-€30.54	-€40.41

In comparing HH2 to the SOC, there is also an increase in costs between the SOC and HH2 (+ €9.50 for Azacetidine and + €12.47 for Bortezomib). This cost difference between HH2 and the SOC is due to the travel time of the nurse to administer the chemo at home. However, there is almost no funding for subcutaneous administration in home care. If the product is administered in a day hospital, the hospital receives revenue of €124.10 per administration, while in home care the funding is €5.30 per visit. As a result, the average revenue per administration decreases substantially (-€67.88 for Azacitidine and -€89.42 for Bortezomib), while the costs increase slightly (+ €9.50 or + €12.47). As a consequence, the average result decreases in HH2 compared to the SOC, with €77.38 for Azacitidine and €101.89 for Bortezomib.

## Discussion

A previous Randomized-Controlled Equivalence Trial [3] focused on the outcomes of HH. This study revealed that the implemented HH models are feasible and safe and that a large majority of patients is highly satisfied with HH and that it has a positive impact on their Quality of Life.

In this study, we focused on the costs. We found that the costs are higher in HH1 than in the SOC (+ €50.42). There is a reduction in costs and staff needed in the hospital by moving the blood draw to the home setting (-€23.84), but the costs in home care are higher (+ €74.26). We also see that the extra revenues in home care (+ €33.58) are insufficient to cover the costs. When we compared HH2 to the SOC, the cost difference between the SOC and HH2 (+ €9.50 for Azacetidine) was smaller than in HH1. This cost difference between HH2 and the SOC is due to the travel time of the nurse to administer the chemo at home. These results are in line with those of King et al. [10], Franken et al. [6], and Rischin and Matthews [14], who concluded that home administration is more expensive than hospital administration.

However, our calculation did not take into account the efficiency gains that can be realized in the longer term. In HH1, as the blood test and the chemotherapy preparation are performed before the patient arrives in the day care clinic, the throughput time can be reduced. This allows better use of the available capacity in terms of beds and seats. The previous Randomized-Controlled Equivalence Trial [3] found that the HH1 model leads to a significant reduction of waiting time before therapy administration at the day care unit by 45% per visit (2 h 36 min ± 1 h 4 min vs. 4 h ± 1 h 4 min,*P* < 0.001). Also in HH2 less capacity of beds and seats is necessary as the administration is performed at home. As a result, the use of beds and seats in the day care oncology unit can be optimized and more patients can be admitted with the same capacity. King et al. [10] concluded that when the demand for chemotherapy exceeded ward capacity by up to 50%, home care could provide a less costly strategy than the expansion of a chemotherapy service in the hospital.

In comparing the revenues with the costs, we see that the current funding from the NHI of HH for oncological patients is insufficient, while reimbursement is a key success factor in the uptake of HH [7]. Also, in the SOC for intravenous treatment the current funding is insufficient.

Our findings are limited by the fact that only three Belgian hospitals and two home care organizations were involved in the HH and we focused on 2 HH models. For the cost calculation, the number of patients and hospitals was limited, which has an impact on transport time and possible efficiency gains in organizing home care. Also, we did not take into account the societal costs. Direct healthcare cost only represent a small proportion of the total societal costs of cancer. Other costs are: (1) direct costs outside the health care sector that can be completely attributed to an illness, like patient travel costs and modification of patients’ home, (2) indirect costs which impact consumption of resources, like production loss due to mortality and morbidity, (3) intangible costs quantified in Value of lost life years and Value of lost Quality of Life and (4) other costs like informal nursing/home care [2]. HH will have an impact on the direct costs outside the health care sector, namely on patients’ travel costs and on other costs, like costs of informal care givers. Further research on efficiency gains and societal costs of home hospitalization is necessary.

Even though the present study was only conducted in one country and included a limited number of patients, it provides new information on costs of HH. Oncological treatments are increasingly designed to be administered subcutaneously and orally, and most developed new drugs have increasingly favorable acute toxicity profiles. The latter allows for treatments to be given for longer period of time and to elderly patients, increasing the need for patient-friendly care pathways on the one hand and the need for day care unit capacity on the other hand. HH might be part of the answer to both of these questions.

## Conclusion

A previous study showed that oncological HH is a feasible and safe alternative for hospital care and improves the Quality of Life. However, costs off HH are higher than the SOC and the current funding from Belgian NHI is insufficient to organize HH. As a result, HH for oncology patient is still limited in Belgium. Reimbursement will be a key success factor in the uptake of HH. Therefore, the government recently reviewed the reimbursement of Home Hospitalization, which will improve the uptake.

### Supplementary Information

Supplementary Material 1.

## Data Availability

The data that support the findings of this study are available on request from the corresponding author. The data are not publicly available due to privacy or ethical restrictions.
