# Supplementary material for: Onco@home: comparing the costs and reimbursement of cancer treatment at home with the standard of care
Source: Arch Public Health. 2024 Jun 24;82:95. doi: 10.1186/s13690-024-01317-1 (PMC11194927; doi:10.1186/s13690-024-01317-1)
Supplement: Supplementary file 1 — Supplementary Material 1. [file 13690_2024_1317_MOESM1_ESM.docx]

## Supplementary information

**Supplementary information – Figure A: Automatic registration form for shadowing**


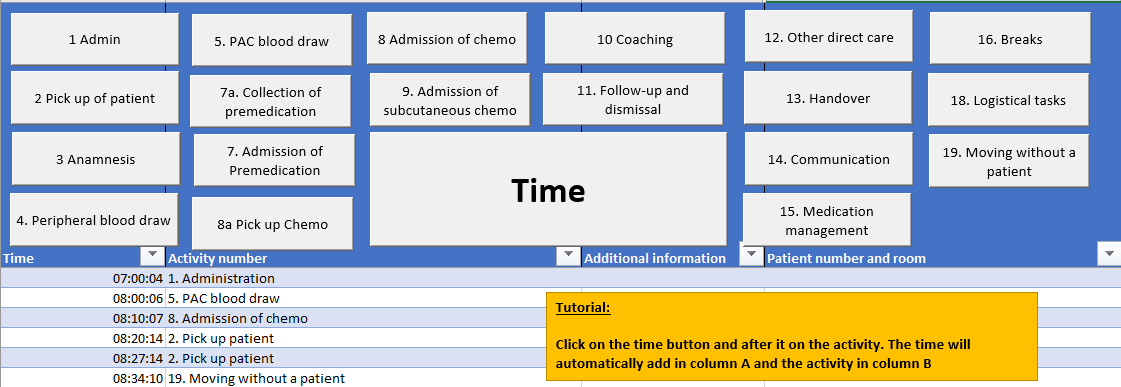


**Supplementary information – Table A: Activities HH1**

| **Activity in home nursing HH1** | **Time in total per day or per patient** |
| --- | --- |
| Administrative preparation of a new patient | In minutes per new patient |
| Planning and calling patients to confirm visit | In minutes per day |
| Travel time to patient | In minutes per patient |
| Blood draw | In minutes per patient |
| Anamnesis, questionnaire on state patient, vital parameters | In minutes per patient |
| Transport of blood samples to hospital | In minutes per day |
| Registration in patient file | In minutes per patient |
| Communication (with General Practitioner, family, …) | In minutes per patient |
| Administration and other tasks | In minutes per day |

**Supplementary information – Table B: Activities HH2**

| **Activity in home nursing HH2** | **Time in total per day or per patient** |
| --- | --- |
| Administrative preparation of a new patient | In minutes per new patient |
| Planning and calling patients to confirm visit, call Medical Doctor, pharmacy, administrative processing | In minutes per day |
| Pick up medication | In minutes per day |
| Travel time to patient | In minutes per patient |
| Admission of subcutaneous chemo | In minutes per patient |

**Supplementary information - Table C: Wage costs per minute**

| **Profile** | **Unit costs**  **(Euro 2019 prices)** | **Source** |
| --- | --- | --- |
| Hospital care: nurse | €0.77 per minute | Wage rates of hospitals (average of the oncology day care units of the three hospitals) |
| Hospital home care nurse HH2 | €0.80 per minute | Wage rates of the hospital for HH2 (only implemented in 1 hospital) |
| Home care: nurse | €0.72 per minute | Wage rates home care (average of two home care organizations) |
| Home care: administrative support | €0.58 per minute | Wage rates home care (average of two home care organizations) |

**Supplementary information – Table D: Time per activity, in minutes, based on time registrations**

|  | **SOC** | | | | | | **HH1** | | | | **HH2** | |
| --- | --- | --- | --- | --- | --- | --- | --- | --- | --- | --- | --- | --- |
| **Type** | **Intravenous treatment 1 product** | | **Intravenous treatment, multiple products** | | **Subcutaneous treatment** | | **Intravenous treatment**  **1 product** | | **Intravenous treatment, multiple products** | | **Subcutaneous treatment**  **At home** | |
| **Number** | **1** | | **2** | | **3 & 4** | | **5** | | **6** | | **7 & 8** | |
| **Patients** | **n = 29** | | **n = 15** | | **n = 2** | | **n = 99** | | | | **n = 15** | |
| **Average time per activity (in minutes)** | **Mean** | **SD** | **Mean** | **SD** | **Mean** | **SD** | **Mean** | **SD** | **Mean** | **SD** | **Mean** | **SD** |
| **Nursing activities per day care visit** | | | | | | | | | | | | |
| Blood draw and symptom control in the hospital | 19,9 | 6.6 | 19.0 | 7.4 | 14.6 | 1.1 | (1) |  | (1) |  | - | - |
| Blood draw + symptom control at home by home nurse | - | - | - | - | - | - | 32.9 | 8.8 | 32.9 | 8.8 | - | - |
| Administration of chemo + follow up | 10.7 | 6.8 | 18.0 | 9.1 | 7.2 | 2.1 | (1) |  | (1) |  |  |  |
| Other direct care time of the nurses in the day care unit | 18.7 | 12.9 | 27.1 | 23.6 | 8.2 | 0.8 | (1) |  | (1) |  |  |  |
| Indirect care time of the nurses in the day care unit | 35.6 | 19.8 | 55.4 | 35.1 | 24.7 | 4.6 | (1) |  | (1) |  |  |  |
| Planning + follow up in home care | - | - | - | - | - | - | 14.6 | 8.4 | 14.6 | 8.4 | - | - |
| **Time hospital (1)** | **84.9** |  | **119.7** |  | **54.7** |  | **65.3** |  | **100.1** |  | **54.7** |  |
| **Time home care** |  |  |  |  |  |  | **47.5** |  | **47.5** |  | **25.9** | **4.4** |

| **Coordination, logistics and administration** | | | | | | | | | | | | |
| --- | --- | --- | --- | --- | --- | --- | --- | --- | --- | --- | --- | --- |
| **Administrative time/visit (2)** | *Calculated as Total cost of coordination, logistics and administration on the day care unit / # day patients* -  - | | | | | | 11.0 | - | 11.0 |  |  |  |
| **Extra administrative time for new patients / total number of visits (16 minutes / 6.5 patients)** |  |  |  |  |  |  | 2.5 |  | 2.5 |  |  |  |
| *Extra administrative time for a new patient (2)* |  |  |  |  |  |  | *8.0* | *-* | *8.0* | - |  |  |
| *Number of new patients per round 2)* |  |  |  |  |  |  | *2.0* |  | *2.0* |  |  |  |
| *Avg. patients/round* |  |  |  |  |  |  | *6.5* | *-* | *6.5* | - |  | - |
| **Total time** |  |  |  |  |  |  | **13.5 (3)** |  | **13.5 (3)** |  | **12.0 (3)** |  |
| *Travel time* |  |  |  |  |  |  |  |  |  |  |  |  |
| **Pick up of the treatment (for HH2)** |  |  |  |  |  |  |  |  |  |  |  |  |
| *Pick up treatment, (in minutes / round)* | - | - | - | - | - | - | - | - | - | - | 7.7 | 2.5 |
| *Avg. # patients/round* | - | - | - | - | - | - | *6.5* | *-* | *6.5* | *-* | *2.0* | *-* |
| **Avg. pick up time/patient** |  |  |  |  |  |  |  |  |  |  | **3.9** |  |
| **Transport to patient** |  |  |  |  |  |  |  |  |  |  |  |  |
| **Transport time to patient** | **-** | **-** | **-** | **-** | **-** | **-** | **14.4** | **6.6** | **14.4** | **6.6** | **16.3** | **8.3** |
| **Transport to hospital** |  |  |  |  |  |  |  |  |  |  |  |  |
| *Transport time to hospital (in minutes / round)* | - | - | - | - | - | - | *16.5* | *4.5* | *16.5* | *4.5* | *22.4* | *6.1* |
| *Avg. # patients/round* | - | - | - | - | - | - | *6.5* | *-* | *6.5* | *-* | *2.0* | *-* |
| **Avg. transport time to hospital per patient** |  |  |  |  |  |  | **2.5** |  | **2.5** |  | **11.2** |  |
| **TOTAL avg. travel time per patient** |  |  |  |  |  |  | **16.9** |  | **16.9** |  | **31.4** |  |

1. In HH1 the blood draw is performed at home. Therefore the weighted average time for blood draw and symptom control in the SOC of 19.6 minutes is subtracted from the total time in the SOC to calculate the care time in the hospital in HH1.
2. These activities remain the same in HH1, as only the blood draw and symptom control is performed at home
3. Added based on interviews; no s.d. available.

**Supplementary information – Table E: Cost of care time nurses in the Standard of Care**

| **Number treatment type** | **1** | **2** | **3** | **4** |
| --- | --- | --- | --- | --- |
| Care time nurses (1) | 84.9 | 119.7 | 54.7 | 54.7 |
| Wage cost per minute (2) | €0.77 | €0.77 | €0.77 | €0.77 |
| **Cost of care time** | **€65.37** | **€92.17** | **€42.12** | **€42.12** |

1. See: supplementary information – table D.
2. See: supplementary information – table C.

**Supplementary information – Table F: Cost of coordination, logistics and administration in the day hospital**

For the administrative, logistic and coordinating staff in the day hospital, the total cost per profile per hospital was requested. To calculate the average cost per patient and per hospital, the cost per year was divided by the total number of day hospital patients.

The detailed calculations can be found here:

**Supplementary information – Table G: Calculation of staff costs HH1 and HH2 in hospital and home care**

**HH1**

Staff costs in hospital

| **Number treatment type** | **7** | **8** |
| --- | --- | --- |
| Care time nurses in hospital (1) | 65.3 | 100.1 |
| Wage cost per minute (2) | €0.77 | €0.77 |
| **Cost of care time** | **€50.28** | **€77.08** |

1. See: supplementary information – table D.
2. See: supplementary information – table C.

Staff costs in home care

| Activity | Time (in minutes) (1) | Cost/minute (in €) (2) | Total cost / activity (in €) |
| --- | --- | --- | --- |
| **Nursing activities** | **47.5** |  |  |
| Blood draw + symptom control at home by a home nurse | 32.9 | 0.72 | 23.69 |
| Planning + follow-up | 14.6 | 0.72 | 10.51 |
| **Coordination, logistics and administration** | **13.5** |  |  |
| Administration and planning | 11.0 | 0.58 | 6.38 |
| Extra time for administration nurse (new patients) | 2.5 | 0.72 | 1.79 |
| **Travel time** | **16.9** |  |  |
| Transport time / patient | 16.9 | 0.72 | 12.17 |
| **TOTAL** |  |  | **54.54** |

1. See: supplementary information – table D.
2. See: supplementary information – table C.

**HH2**

| **Treatment type number** | **7** | **8** |
| --- | --- | --- |
| Care time nurses in hospital per visit (1) | 54.7 | 54.7 |
| Wage cost per minute hospital nurse | €0.77 | €0.77 |
| Number of visits in hospital | 3 | 1 |
| **Total staff cost in hospital** | **€42.12** | **€42.12** |
| Care time nurses in home care (1) | 69,3 | 69,3 |
| Wage cost per minute – home admission by hospital nurse | €0.80 | €0.80 |
| Number of visits at home | 4 | 3 |
| **Total staff cost at home** | **€55.44** | **€55.44** |
|  |  |  |
| Total number of visits | 7 | 4 |
| **Average cost in hospital** | **€18.05** | **€10.53** |
| **Average cost at home** | **€31.68** | **€41.58** |

1. See: supplementary information – table D.
2. See: supplementary information – table C.

**Supplementary information – Table H: Material costs**

| **Type of cost** | **Cost per patient visit** | **Source** |
| --- | --- | --- |
| Material for blood draw in hospital | €8.76 | Financial administration of two hospitals (Average) |
| Material for blood draw at home (paid by the hospitals in the pilot) | €9.00 | Financial administration of two hospitals (Average) |
| Cost of other materials in home care (2) | €0.21 | (2) |
| Material for administration of one chemo product/day | €1.37 | Financial administration of two hospitals (Average) |
| Material of administration of multiple chemo products/day (1) | €6.51 | Financial administration of two hospitals (Average) |
| Material for administration of subcutaneous treatment | €0.41 | Financial administration of two hospitals (Average) |

1. Assuming two products per administration, the number of products per administration can also be three or four.
2. The home care organizations registered also the costs of other materials used: laptop, phone, balance, thermometer, … These costs were divided by the total number of visits per year and per home nurse. In hospitals, these costs are included in the overhead percentage, while this was not included in the overhead percentage of home care.

Cost of other material in home care:

|  | Cost per visit |
| --- | --- |
| Depreciation cost computer | €0.093 |
| Depreciation cost phone | €0.015 |
| Balance | €0.009 |
| Thermometer | €0.003 |
| Blood pressure monitor | €0.017 |
| Medical waste container | €0.005 |
| Compression band | €0.049 |
| Needle holder | €0.001 |
| Disinfector | €0.000 |
| Printer | €0.022 |
| **TOTAL** | **€0.214** |

**Supplementary information – Table I: Average material cost per care pathway**

This calculations are based on the material costs in Table H.

|  | **SOC** | | | | **HH1** | | **HH2** | |
| --- | --- | --- | --- | --- | --- | --- | --- | --- |
| **Nr** | **1** | **2** | **3** | **4** | **5** | **6** | **7** | **8** |
| Blood draw | €8.76 | €8.76 | €1.25 (1) | €2.19  (1) | €9.00 | €9.00 | €1.25 (1) | €2.19  (1) |
| Other materials in home care |  |  |  |  | €0.21 | €0.21 |  |  |
| Administration of chemo (2) |  |  |  |  |  |  |  |  |
| - At home |  |  |  |  |  |  | €0.41 | €0.41 |
| - In hospital | €1.37 | €6.51 | €0.41 | €0.41 | €1.37 | €6.51 |  |  |
| **Average cost in hospital** | **€10.13** | **€15.27** | **€1.66** | **€2.60** | **€1.37** | **€6.51** |  |  |
| **Average cost at home** |  |  |  |  | **€9.21** | **€9.21** |  |  |
| **Average cost** |  |  |  |  |  |  | **€1.66** | **€2.60** |

1. Average cost per cycle: Care Pathway 3 and 7: Average cost of one visit, 7 administrations of chemo per cycle, one blood test per cycle, performed in the hospital = €8.76/7 = €1.25; Care Pathway 4 and 8: Average cost of one visit, four visits per cycle, one blood test per cycle = €8.76/4 = €2.19
2. Price is equal in home care and in hospital

**Supplementary information – Table J: Car costs per visit**

To calculate the car costs, the nurses registered the distance to each patient during the time registration of HH1 and HH2. Based on this information, an average distance per patient was calculated. Per km an average cost of €0.35 was used, based on the kilometer allowance defined by the government (Federale Overheidsdienst Beleid en Ondersteuning, 2020). The average distance in HH1 was 9.46 km (+/-3.9 km SD) and in HH2 11.33 km (+/- 6.4 km SD).

|  | **HH1** | **HH2** | |
| --- | --- | --- | --- |
|  | **Intravenous treatment 1 or multiple products** | **Azacetidine**  **3 visits in hospital, 4 at home** | **Bortezomib**  **1 visit in hospital, 3 at home** |
| Average number of kilometers | 9.46 km | 11.33 km | 11.33 km |
| Cost per km | €0.35 | €0.35 | €0.35 |
| Car cost per visit at home | €3.31 | €3.31 | €3.31 |
| Car cost per care pathway (car cost per visit * number of visits at home per cycle) | €3.31 | €13.24 | €9.93 |
| Total number of admissions in the calculation (in hospital + at home) | 1 | 7 | 4 |
| **Average car cost per admission** | **€3.31** | **€1.89** | **€2.48** |

**Supplementary information – Table K: Revenues per visit**

| Revenues per visit (in minutes) | SOC – Administration in hospital | | | HH Model 1  Blood draw and symptom control at home | | HH Model 2 |
| --- | --- | --- | --- | --- | --- | --- |
|  | Intravenous treatment  1 product | Intravenous treatment, multiple products | Subcutaneous treatment | Intravenous treatment  1 product | Intravenous treatment, multiple products | Subcutaneous treatment  At home |
| Hospital | €124.10 | €166.11 | €124.10 | €124.10 | €166.11 | €124.10 |
| Home care |  |  |  | €33.58 (1) | €33.58 (1) | €5.30 (2) |

1. For blood draw and symptom control at home, the admission of the intraveneous treatment is performed in the hospital the next day.
2. For the admission of the subcutaneous treatment at home
